# Nonstructural 5A Protein of Hepatitis C Virus Interferes with Toll-Like Receptor Signaling and Suppresses the Interferon Response in Mouse Liver

**DOI:** 10.1371/journal.pone.0170461

**Published:** 2017-01-20

**Authors:** Takeya Tsutsumi, Kazuya Okushin, Kenichiro Enooku, Hidetaka Fujinaga, Kyoji Moriya, Hiroshi Yotsuyanagi, Hideki Aizaki, Tetsuro Suzuki, Yoshiharu Matsuura, Kazuhiko Koike

**Affiliations:** 1 Department of Internal Medicine, Graduate School of Medicine, The University of Tokyo, Tokyo, Japan; 2 Department of Virology II, National Institute of Infectious Diseases, Tokyo, Japan; 3 Department of Infectious Diseases, Hamamatsu University School of Medicine, Hamamatsu, Japan; 4 Department of Molecular Virology, Research Institute for Microbial Diseases, Osaka University, Osaka, Japan; Saint Louis University, UNITED STATES

## Abstract

The hepatitis C virus nonstructural protein NS5A is involved in resistance to the host immune response, as well as the viral lifecycle such as replication and maturation. Here, we established transgenic mice expressing NS5A protein in the liver and examined innate immune responses against lipopolysaccharide (LPS) *in vivo*. Intrahepatic gene expression levels of cytokines such as interleukin-6, tumor necrosis factor-α, and interferon-γ were significantly suppressed after LPS injection in the transgenic mouse liver. Induction of the C-C motif chemokine ligand 2, 4, and 5 was also suppressed. Phosphorylation of the signal transducer and activator of transcription 3, which is activated by cytokines, was also reduced, and expression levels of interferon-stimulated genes, 2’-5’ oligoadenylate synthase, interferon-inducible double-stranded RNA-activated protein kinase, and myxovirus resistance 1 were similarly suppressed. Since LPS binds to toll-like receptor 4 and stimulates the downstream pathway leading to induction of these genes, we examined the extracellular signal-regulated kinase and IκB-α. The phosphorylation levels of these molecules were reduced in transgenic mouse liver, indicating that the pathway upstream of the molecules was disrupted by NS5A. Further analyses revealed that the interaction between interleukin-1 receptor-associated kinase-1 and tumor necrosis factor receptor associated factor-6 was dispersed in transgenic mice, suggesting that NS5A may interfere with this interaction via myeloid differentiation primary response gene 88, which was shown to interact with NS5A. Since the gut microbiota, a source of LPS, is known to be associated with pathological conditions in liver diseases, our results suggest the involvement of NS5A in the pathogenesis of HCV infected-liver via the suppression of innate immunity.

## Introduction

Hepatitis C virus (HCV) is one of the most prevalent infectious diseases worldwide. Chronic infection develops in the majority of patients, often leading to liver cirrhosis and hepatocellular carcinoma (HCC) [1]. The HCV genome is a single-stranded positive RNA molecule encoding viral structural proteins such as core, E1, E2, and the p7 protein, and non-structural (NS) proteins such as NS2, NS3, NS4A, NS4B, NS5A, and NS5B. The NS5A is a multifunctional phosphoprotein consisting of 447 amino acids residues. In addition to the association with the viral lifecycle, such as replication and maturation [2,3], NS5A is known to have pleiotropic effects on host cells, including regulation of host gene expression and cellular signaling pathways [4]. NS5A is also implicated in antiviral resistance to interferon (IFN) [5]. NS5A contains an IFN sensitivity-determining region (ISDR) and interacts with the IFN-inducible double-stranded RNA-activated protein kinase (PKR) through the ISDR, leading to the repression of PKR activity [6]. NS5A also attenuates IFN action by inducing expression and secretion of interleukin (IL)-8, a pro-inflammatory chemokine that interferes with IFN through alterations in IFN-stimulated genes (ISG) [7]. NS5A is also known to directly inhibit IFN-αsignaling by abrogating signal transducer and activator of transcription (STAT)-1 phosphorylation and nuclear translocation [8].

Toll-like receptors (TLRs) are a class of proteins that play a key role in the innate immune system. They are membrane-spanning, non-catalytic receptors that recognize structurally conserved molecules derived from microbes. More than 10 different TLRs have been identified [9]. Once a TLR is stimulated by pathogen-associated molecules, intracellular signaling cascades are activated, culminating in the induction of multiple genes. TLR signaling is mediated primarily by the adaptor protein myeloid differentiation factor 88 (MyD88), which triggers activation of downstream signaling molecules such as IL-1 receptor-associated kinase (IRAK) and tumor necrosis factor (TNF) receptor associated factor-6 (TRAF6). These events are followed by activation of mitogen-activated protein kinase (MAPK) and nuclear factor-κB (NF-κB), which are essential for the expression of pro-inflammatory cytokine genes.

The innate immune system is important as a primary host defense system against microbes, and HCV is known to disrupt this system [10–12]. We previously reported that in a macrophage cell line, HCV NS5A protein impairs cytokine reduction in response to TLR ligands by directly interacting with MyD88, a major adapter molecule in the TLR signaling pathway [13].

Therefore, we examined whether HCV NS5A protein modulates TLR signaling *in vivo* using transgenic mice. Similar to the data derived from our previous *in vitro* study, NS5A impaired the activation of TLR signaling pathways in response to lipopolysaccharide (LPS), leading to the suppression of cytokine expression and ISGs in the liver. These results suggest that HCV NS5A may facilitate viral replication by protecting viruses against the immune system and contribute to liver pathogenesis by HCV.

## Materials and Methods

### Transgenic mice

Transgenic mice harboring the HCV NS5A gene (HCV genotype 1b, mouse strain C57BL/6 from Charles River Laboratories) were generated in the same way as HCV core-transgenic or envelope-transgenic mice [13–15]. Briefly, the NS5A gene of HCV, which is placed downstream of a transcriptional regulatory region from hepatitis B virus, was introduced into mouse embryos. Mice, 3 to 5 mice per cage, were maintained in a specific, pathogen-free facility on a 12-h light/12-h dark cycle at 24°C with free access to water and standard diet (CLEA Japan, Tokyo, Japan). The expression of NS5A was confirmed by immunoblotting with an anti-NS5A monoclonal antibody (Millipore, Billerica, MA). Levels of NS5A mRNA were quantified by real-time polymerase chain reaction (PCR) with a specific probe and primers (Applied Biosystems, Foster City, CA). For histological examination, tissues sections (5 μm thick) fixed in 10% neutral-buffered formalin were used for hematoxylin and eosin (H&E) staining. This study was approved and carried out in strict accordance with the recommendations by Laboratory of Animal Resources in Faculty of Medicine, The University of Tokyo. Mice were sacrificed by cervical dislocation with all efforts to minimize suffering.

### Administration of LPS to mice

Eight pairs of 6-month-old male transgenic and nontransgenic littermates were prepared for the study. LPS (Sigma-Aldrich, St. Louis, MO) was dissolved in normal saline and injected intraperitoneally (1 μg/g body weight) into five pairs of transgenic and nontransgenic mice. As a control, the same volume of normal saline was injected into three pairs of mice. After 6 h of injection, mice were sacrificed and sera and livers were obtained. Levels of serum aspartate aminotransferase (AST) and alanine aminotransferase (ALT) were measured using the DRI-CHEM 3000 V (Fujifilm, Tokyo, Japan). No mice died or became severely ill during the experiment, although we planned to euthanize mice if they struggled madly from pain or lost mobility.

### Immunoblotting, immunoprecipitation, and immunohistochemistry

Liver tissues of mice were lysed in NET-N buffer (20 mM Tris-HCl [pH 8.0], 100 mM NaCl, 1 mM EDTA, 0.5% NP-40) containing protease inhibitor cocktail set I (Merck Millipore, Darmstadt, Germany), and lysates were subjected to immunoblot analysis. For immunoprecipitation, cell lysates were incubated with an anti-TRAF6 antibody (Poteintech, Rosemont, IL) and protein A-Sepharose beads (GE Healthcare, Uppsala, Sweden) overnight and then washed vigorously three times with lysis buffer. Antibodies against STAT3, phosphorylated STAT3, extracellular signal-regulated kinase (ERK), phosphorylated ERK, IκB-α, phosphorylated IκB-α, and phosphorylated IFN regulatory factor (IRF)-7 were purchased from Cell Signaling Technologies (Danvers, MA). Anti-IRAK1 and anti-IRF-7 antibodies were purchase from Santa Cruz Biotechnology (Dallas, TX), and anti-vinculin and anti-glyceraldehyde-3-phosphatate dehydrogenase (GAPDH) antibodies were purchased from Merck Millipore. For immunohistochemistry, liver tissue sections were incubated with an anti-phospho-STAT3 antibody followed by detection using HISTOFINE SimpleStain Mouse MAX-PO(R) (Nichirei Biosciences Inc., Tokyo, Japan).

### Quantitative real-time PCR

Total RNA was extracted from livers of mice using a SV Total RNA Isolation System (Promega, Madison, WI). Three micrograms of RNA was reverse transcribed using a High Capacity cDNA Reverse Transcription Kit (Applied Biosystems). Specific cDNA was quantified using the real-time PCR 7000 with a TaqMan Gene Expression Master Mix and specific probe/primers. As an internal control, mouse β-actin gene was amplified with specific probe/primers (Applied Biosystems).

### Electrophoretic mobility shift assay (EMSA)

Nuclear extracts were prepared from liver tissue of the mice using NE-PER Nuclear and Cytoplasmic Extraction Reagents (Pierce, Rockford, IL). EMSA was performed using LightShift Chemiluminescent EMSA Kit (Pierce). Briefly, biotin-labeled oligonucleotides containing STAT3-binding site (5’-GAT CCT TCT GGG AAT TCC TAG ATC-3’) were incubated on ice for 20 minutes along with 10 μg of nuclear proteins in binding buffer, and were then subjected to electrophoresis with 6% polyacrylamide native gel in 0.5X Tris/borate/EDTA. To confirm the specificity of the shifted band, 100-fold amount of non-labeled, identical or mutated (5’-GAT CCT TCT GGG CCG TCC TAG ATC-3’) oligonucleotides were incubated with nuclear proteins 20 min before the addition of labeled oligonucleotides.

### Statistical analysis

Results are expressed as means ± SE. The significance of the difference was determined based on the Mann-Whitney *U*-test.

## Results

### Generation of HCV NS5A gene transgenic mice

**Fig 1:**
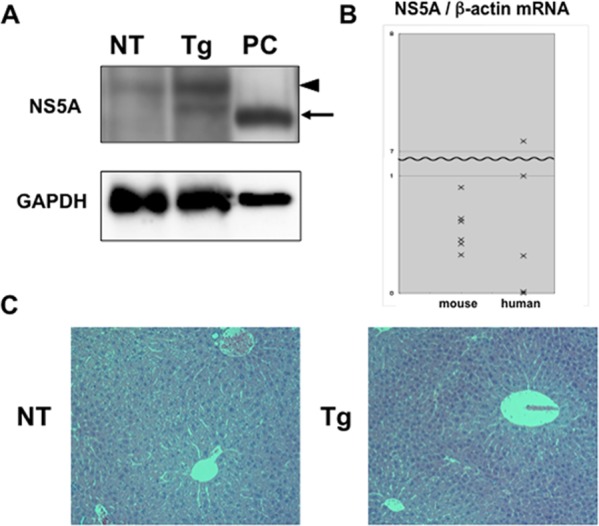
Expression of NS5A in transgenic mice. (A) Expression levels of NS5A protein in liver tissues of NS5A-transgenic (Tg) and non-transgenic (NT) mice were determined by immunoblotting with mouse anti-NS5A antibody. As a positive control (PC), lysates of the HCV subgenomic replicon cells were used. The arrow represents the NS5A protein. The arrowhead indicates a nonspecific band observed only in mouse tissues. (B) mRNA expression levels of NS5A in the liver of transgenic mice and HCV-infected patients were determined using quantitative real-time PCR. The levels were standardized to the β-actin gene. The median value of human samples was defined as 1 in Y-axis. (C) Liver tissue sections of the 6-month-old NS5A-transgenic (Tg) and non-transgenic (NT) mice were histologically examined with H&E staining.

To examine the role of NS5A in HCV-induced pathogenesis *in vivo*, transgenic mice stably expressing NS5A protein in the liver were established. The NS5A gene from HCV genotype 1b was expressed under the control of regulatory elements from hepatitis B virus in these transgenic mice, similar to HCV core-transgenic mice reported previously [14,15]. The expression of NS5A was analyzed by immunoblotting and a specific band corresponding to 56 kDa was detected in the transgenic mouse liver, although the mobility of NS5A protein was slightly different between the transgenic mice and Huh7 cells harboring HCV subgenomic replicon, probably due to the difference in phosphorylation (Fig 1A) [16,17]. However, the level of NS5A expression was very low compared to replicon cells. Probably for this reason, we could not detect specific NS5A expression by immunohistochemistry in liver tissue sections of the transgenic mice, although we tried several conditions. To compare expression levels of NS5A, we extracted RNA from livers of transgenic mice and HCV-infected patients and quantified NS5A RNA expression using real-time PCR. As shown in Fig 1B, RNA expression levels of NS5A in mice were not distinct from those of human livers, although the levels ranged widely in human samples. To explore whether NS5A expression induces any phenotypes *in vivo*, we bred transgenic mice and sacrificed them at several ages up to 24 months, but no significant histological changes were observed in livers or other organs (Fig 1C).

### Suppressed cytokine/chemokine induction in NS5A-transgenic mice

**Fig 2:**
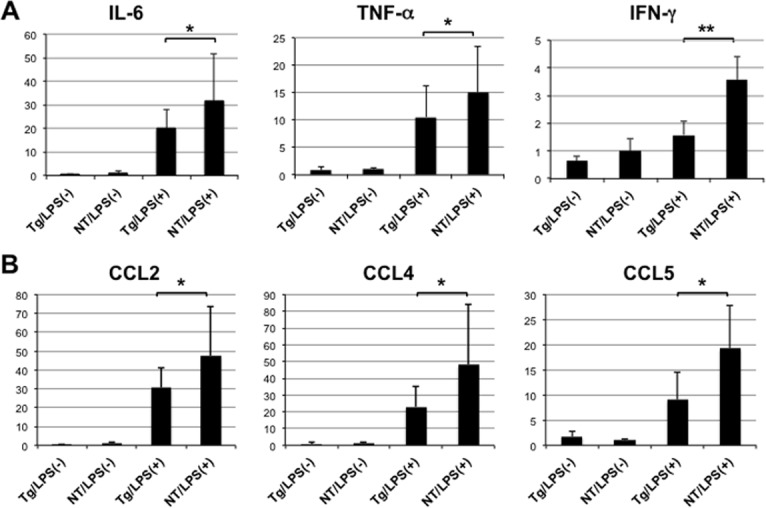
Expression of intrahepatic cytokines and chemokines. (A) Expression levels of IL-6, TNF-α, and IFN-γ genes were determined using quantitative real-time PCR in the livers of NS5A-transgenic (Tg) and non-transgenic (NT) mice 6 h after injection with LPS (N = 5, each) or normal saline (N = 3, each). (B) Expression levels of CCL2, CCL4, and CCL5 were similarly determined using quantitative real-time PCR. * *p*<0.05, ** *p*<0.01.

Since NS5A is known to be associated with the host immune response *in vitro* and *in vivo*, we administered LPS intraperitoneally to five pairs of the transgenic and nontransgenic mice and examined the effect on the innate immune response in the liver by sacrificing the mice 6 hours after injection. As a control, three pairs of mice were injected with normal saline. All of the mice looked apparently healthy during the study regardless of LPS injection. We first determined mRNA expression levels of inflammatory cytokines using real-time PCR with specific primers/probe. As shown in Fig 2A, expression levels of IL-6, TNF-α, and IFN-γ increased after LPS administration, but the induction levels were low in transgenic mice compared to nontransgenic mice. We also examined intrahepatic mRNA levels of chemokines such as C-C motif chemokine ligand (CCL) 2 (also called monocyte chemotactic protein-1; MCP-1), CCL4 (macrophage inflammatory protein-1β; MIP-1β), and CCL5 (regulated on activation, normal T cell expressed and secreted; RANTES). As shown in Fig 2B, induction levels of these CCL chemokines by LPS were reduced in livers of transgenic mice.

### Impairment of STAT3 activation and ISG induction by LPS in NS5A-transgenic mice

**Fig 3:**
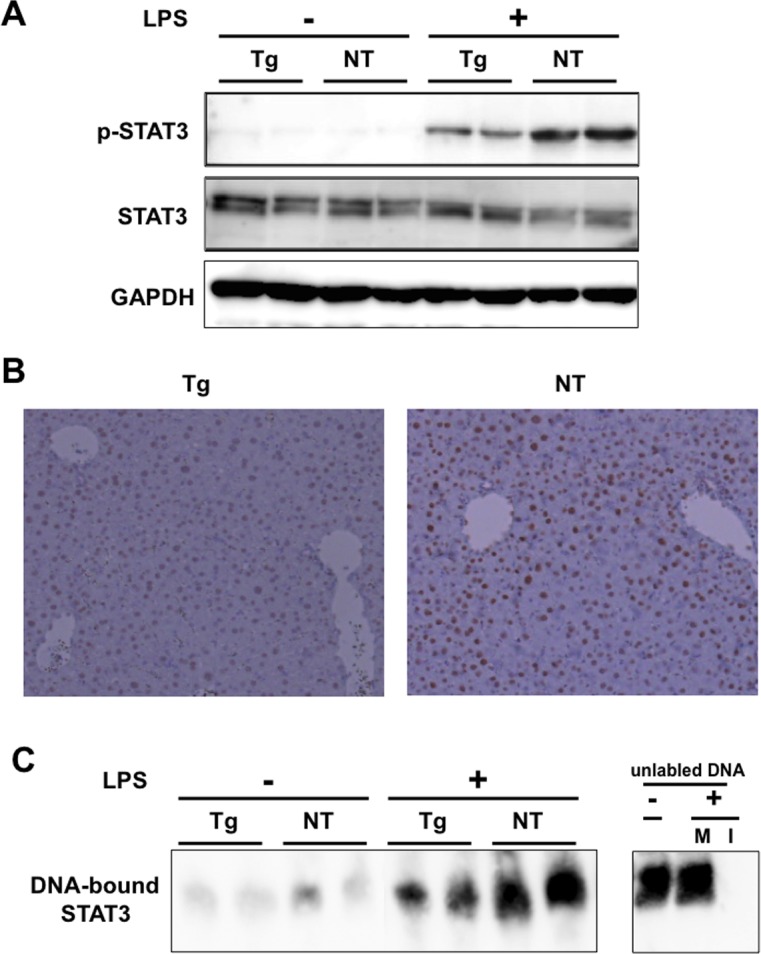
Suppressed activation of STAT3 in NS5A-transgenic mice. (A) Phosphorylation levels of STAT3 were determined by immunoblotting with phospho-specific antibodies in the livers of NS5A-transgenic (Tg) and non-transgenic (NT) mice 6 h after injection with LPS or normal saline. (B) Liver tissue sections derived from LPS-injected transgenic (Tg) and nontransgenic (NT) mice were immunohistologically stained with an anti-phospho-STAT3 antibody. (C) DNA-binding activity of STAT3 was determined by EMSA. Shifted bands corresponding to DNA-bound STAT3 were shown (left panel). The specificity of the band was examined by adding 100-fold amount of non-labeled, identical (I) or mutated (M) oligonucleotides before the addition of labeled oligonucleotides (right panel).

Since cytokines such as IL-6 and IFN activate STAT3 by phosphorylation, we next examined the phosphorylation status of STAT3 by LPS in livers of the mice. In mice inoculated with saline, levels of phosphorylated STAT3 were similarly low in both transgenic and nontransgenic mice. In mice with LPS, phosphorylated STAT3 was increased, but the phosphorylation levels were decreased in transgenic mice compared to nontransgenic mice (Fig 3A). Immunostaining of mouse liver tissues revealed decreased expression levels of phosphorylated STAT3 mainly in hepatocytes of LPS-injected NS5A transgenic mouse compared to nontransgenic mouse (Fig 3B). On the other hand, no expression was detected in mice without LPS (S1 Fig). We also performed EMSA to examine the DNA-binding activity of STAT3 and found that DNA-bound STAT3 was decreased in transgenic mice (Fig 3C).

**Fig 4:**
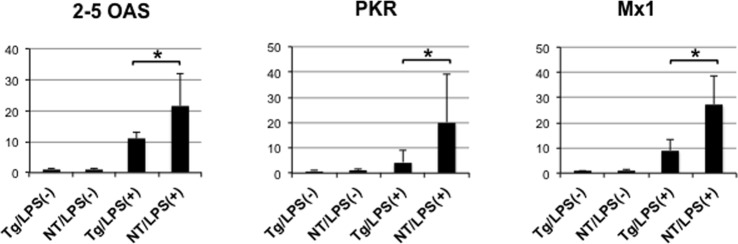
Suppressed expression of ISGs in NS5A-transgenic mice. Expression levels of 2–5 OAS, PKR, and Mx1 genes were determined by quantitative real-time PCR in the livers of NS5A-transgenic (Tg) and non-transgenic (NT) mice injected with LPS (N = 5, each) or normal saline (N = 3, each). * *p*<0.05.

We next examined expression levels of 2’-5’ oligoadenylate synthase (2–5 OAS), IFN-inducible double-stranded RNA-activated PKR, and myxovirus resistance 1 (Mx1), which are known ISGs. In livers of saline-injected mice, levels of mRNA expression of these genes were very low (Fig 4). In LPS-injected nontransgenic mice, expression levels of these genes increased about 20-fold. However, transgenic mice treated with LPS showed a significantly decreased induction compared to nontransgenic mice. The levels were from a half to a quarter of those in nontransgenic mice. These results showed that upregulation of ISGs was suppressed in livers of NS5A-transgenic mice, suggesting that NS5A disrupts the IFN-induced immune response *in vivo*.

**Fig 5:**
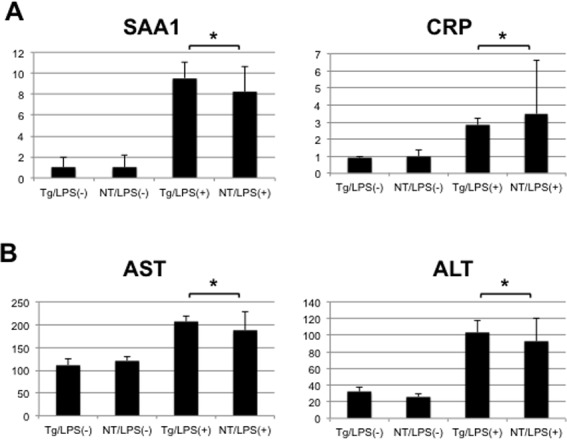
Expression of genes related to inflammatory response, and serum levels of transaminases in mice. (A) Expression levels of SAA1 and CRP genes were determined using quantitative real-time PCR in the livers of NS5A-transgenic (Tg) and non-transgenic (NT) mice 6 h after injection with LPS (N = 5, each) or normal saline (N = 3, each). (B) Serum levels of AST and ALT were determined in these mice using a dry chemistry technique. * statistically not significant.

The results showed that expression of intrahepatic cytokines and ISGs was suppressed in LPS-injected transgenic mice; thus, we explored differences in the inflammatory reaction in mice based on mRNA expression levels of C-reactive protein (CRP) and serum amyloid A1 (SAA1), which are acute phase proteins induced by inflammation in the livers of these mice. As shown in Fig 5A, expression of these genes was induced by LPS injection, but the levels were similar between transgenic and nontransgenic mice. Since intrahepatic inflammation causes hepatocyte injury, leading to increases in hepatic transaminases such as AST and ALT, we also examined serum levels of these enzymes in mice. The levels of AST and ALT were induced by LPS by about 2-fold and 3-fold, respectively, but there were no significant differences between LPS-injected transgenic and nontransgenic mice (Fig 5B). In fact, the findings based on pathological examination of liver tissues with H&E staining were similar between these mice (data not shown). These results suggested that inflammatory responses against LPS were not different in NS5A-transgenic mice, despite the differences in innate immune responses, at least at the dosage used in this study.

### Impaired TLR4 signaling pathway by NS5A protein

**Fig 6:**
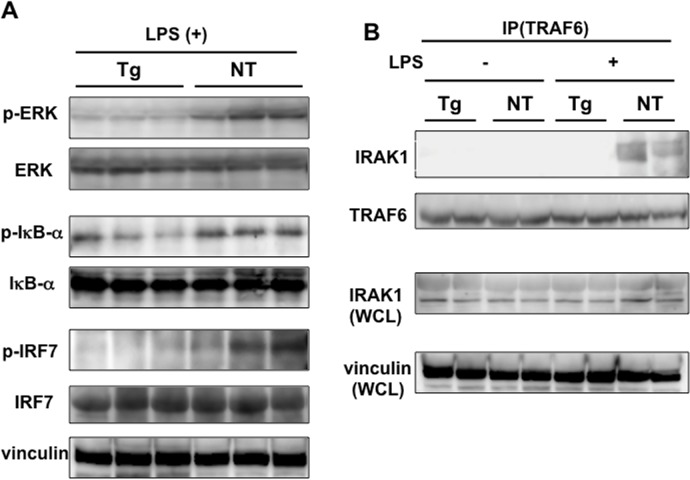
Suppressed TLR signaling in the livers of LPS-injected NS5A-transgenic mice. (A) Phosphorylation levels of ERK, IκB-α, and IRF7 were determined by immunoblotting with phospho-specific antibodies in the livers of NS5A-transgenic (Tg) and non-transgenic (NT) mice injected with LPS. (B) Interaction between IRAK1 and TRAF6 was examined using an immunoprecipitation experiment. Liver tissue lysates of NS5A-transgenic (Tg) and non-transgenic (NT) mice were immunoprecipitated with anti-TRAF6 antibody and the immunoprecipitates were subjected to SDS-PAGE followed by immunoblotting with anti-IRAK1 and anti-TRAF6 antibodies. Whole cell lysates (WCL) were similarly subjected to immunoblotting with anti-IRAK1 and anti-vinculin antibodies.

The above results suggested that HCV NS5A suppresses LPS-induced intrahepatic cytokine production and STAT3 activation *in vivo*, and that this suppression occurred through the TLR signaling pathway since LPS binds to the TLR4 receptor and activates the downstream pathway, leading to cytokine induction. The intracellular TLR signaling pathway includes ERK, NF-κB, and IRF, upstream regulators controlling expression levels of cytokine genes. Therefore, we explored the activation of these molecules by LPS stimulation in mice. As shown in Fig 6A, levels of phosphorylated ERK were lower in transgenic mice than in non-transgenic mice. IκB-α is an inhibitor of NF-κB and phosphorylation of IκB-αleads to its degradation and activation of NF-κB. Levels of phosphorylated IκB-αwere similarly lower in transgenic mice, indicating that NF-κB activation was also suppressed. In addition, IRF7 was similarly less phosphorylated in transgenic mice. These results indicated that activation of intracellular TLR4 signaling by LPS is suppressed in transgenic mice, possibly due to the inhibition of molecules upstream of ERK, IκB-α, and IRF7 by NS5A.

Since transforming growth factor β-activated protein kinase-1 (TAK1), an upstream regulator of ERK and NF-κB, is activated by the interaction of IRAK1 with TRAF6, we performed an immunoprecipitation experiment to examine this interaction. In the liver of saline-injected transgenic and non-transgenic mice, no interaction was observed. In LPS-injected non-transgenic mice, co-immunoprecipitation of IRAK1 with an anti-TRAF6 antibody was observed (Fig 6B), indicative of the TRAF6-IRAK1 interaction through activation of TLR4 signaling by LPS stimulation. In transgenic mice with LPS, this interaction was almost absent, suggesting that NS5A directly or indirectly interferes with the interaction. The specificity of the interaction was confirmed by the immunoprecipitation with control IgG (S2 Fig). These results suggested that NS5A suppresses the TLR signaling pathway by disturbing the TRAF6/IRAK1 interaction *in vivo*.

## Discussion

In this study, we generated transgenic mice expressing HCV NS5A protein in the liver and found that the mice have impaired TLR4 signaling. When the mice were injected with LPS, which binds to a cell surface receptor TLR4 and activates the TLR signaling pathway, suppressed activation of STAT3 and reduced induction of intrahepatic cytokines and ISGs were observed. The mechanism of impaired TLR signaling by NS5A is the inhibition of the direct interaction between IRAK1 and TRAF6. We previously reported that NS5A binds to MyD88, a major adaptor molecule in TLR, which inhibits the recruitment of IRAK1 to MyD88 in response to TLR ligands [13]. Since the expression levels of NS5A in our transgenic mice are as low as those in human infected livers, the interaction of NS5A with MyD88 could not be detected (data not shown), but the observation that the interaction between IRAK1 and TRAF6 was impaired in transgenic mice suggests that NS5A may interact with MyD88 in the liver of transgenic mice.

LPS is a large molecule consisting of a lipid and polysaccharide, and a major component of the outer membrane of Gram-negative bacteria. Recently, the interaction between the liver and gut as an immune defense system, called the gut-liver axis, has been examined [18,19]. The liver hosts the entire spectrum of the immune cell repertoire and has a remarkable capacity to recruit and activate immune cells in response to gut-derived metabolic or pathogen-derived signals. The effect of gut microbiota has been of major interest in liver diseases such as alcoholic liver diseases (ALD) and non-alcoholic fatty liver diseases (NAFLD) [20–23]. In ALD patients, levels of bacterial endotoxin were increased in portal circulation, which may have been caused by disruption of the intestinal epithelial barrier by alcohol consumption, leading to increased gut permeability [20]. NAFLD dysbiosis caused by changes in the gut microbiota is closely associated with certain food components and lifestyle factors, and increases gut permeability to bacterial products, in turn leading to hepatic exposure to injurious substances and subsequent hepatic inflammation and fibrosis [22]. It is possible that LPS may contribute to pathogenesis in the livers of HCV-infected patients, although no studies have demonstrated this association. If NS5A suppresses innate immune responses against LPS, the liver is continuously exposed to the toxicity of LPS and consequently liver damage will be accelerated. In view of this point, we chose 1 μg/g BW of LPS, a relatively low level for administration to mice. Furthermore, attenuation of the immune response may help the virus to replicate efficiently, which could play a role in advanced liver diseases.

Further studies are required based on the results of our study. First, we demonstrated that NS5A suppressed cytokine expression via the TLR signaling pathway, but the cell types affected remain unknown. Cytokines and chemokines are excreted mainly from Kupffer and dendritic cells in the liver; therefore, it is possible that suppression of the innate immune response by NS5A occurred in these cells. However, NS5A protein is mainly localized in hepatocytes, similar to the core protein in transgenic mice, although we could not demonstrate the localization of NS5A using immunohistochemistry due to low expression levels. Therefore, hepatocytes may also be the main targets for immunosuppression. The result that the differential expression of phospho-STAT3 was observed mainly in hepatocytes by immunohistochemistry (Fig 3B) also supports this hypothesis. In addition, hepatocytes are known to express several cytokines/chemokines such as TNF-α and RANTES [24]. Second, we showed that the intrahepatic inflammatory reaction against LPS in transgenic mice did not differ compared to that of non-transgenic mice, while expression levels of cytokines/chemokines and ISGs were suppressed. The reason for this discrepancy remains unclear, but it is possible that the dosage of LPS used in this study is not appropriate to discriminate inflammatory changes between the mice. It is also possible that some mechanisms to inhibit the induction of inflammation by NS5A may be present in transgenic mice. However, further studies are required to explore these hypotheses.

To date, several groups have established and analyzed HCV NS5A transgenic mice, although their strategies, such as the mouse strains and promoter genes used, have differed [25–29]. Similar to our mice, the majority of NS5A-transgenic mice reported previously were phenotypically normal, except for one report by Wang et al. showing that NS5A transgenic mice developed steatosis and HCC with no special treatments [29]. Since NS5A was shown to be associated with the IFN response *in vitro*, previous studies have mostly focused on the immune response. Kriegs et al. analyzed NS5A transgenic mice and showed that NS5A bound to PKR and suppressed the induction of IFN-β, 2,5-OAS, and PKR in response to lymphocytic choriomeningitis virus infection [27]. On the other hand, Kanda et al. showed that IFN-γ expression after adenoviral infection was inhibited in NS5A transgenic mice, contributing to failure of early viral clearance, whereas STAT3 was constitutively activated in their mice, as reported previously [26,30]. Our results are consistent with the above two studies in that NS5A impairs the innate immune response, although activation of STAT3 was different. However, the mechanism of the downregulation differs from these previous reports. This may be explained by the difference in reagents used and the downstream pathway examined. We used LPS as a stimulant in this study, since LPS is biologically inherent and continuously flowing into the liver. As a result, we found that the TLR4 signaling pathway and its downstream cytokines and ISGs were suppressed by the reduced interaction between IRAK1 and TRAF6. The inhibition of the interaction is probably due to the direct interaction of NS5A with MyD88, which was shown previously in our *in vitro* study [13], although that interaction could not be demonstrated *in vivo*. Another explanation for the different mechanism may be the difference in expression levels of NS5A in transgenic mice. Our transgenic mice have a very low level of NS5A expression, which is barely detectable by immunoblotting, as shown in Fig 1A. This level is almost comparable to those in the livers of HCV-infected patients when we examined human liver samples derived from hepatic surgery (Fig 1B). In addition, the difference of HCV genotypes used to generate NS5A-transgenic mice may account for the difference of the phenotype. We and two studies [27, 29] used mice transgenic for NS5A gene from genotype 1b, whereas the others [25,26,28] used genotype 1a. In this point, to examine the difference in MyD88-NS5A interaction, we performed a co-immunoprecipitation assay using NS5A-expression plasmids derived from genotype 1a and 1b as well as 2a, but all NS5A similarly interacted with MyD88 (S3 Fig), which was also reported previously [13]. It is possible that other mechanisms dependent on HCV genotypes may be involved, and further studies are necessary to elucidate the difference of the mouse phenotype between genotype 1a and 1b.

In conclusion, we showed that transgenic mice expressing HCV NS5A have impaired TLR4 signaling and subsequent decreased expression of cytokines and ISGs in response to LPS. Further analysis revealed that inhibition of TLR4 signaling was caused by a reduced interaction between IRAK1 and TRAF6 and subsequent suppression of ERK and NF-κB activation. Since gut microbiota are strongly associated with liver diseases, these findings suggest that the suppression of innate immunity against LPS by NS5A may be responsible for the pathogenesis in HCV-infected patients. However, further studies are required to predict whether the gut-liver axis actually contributes to the clinical course of chronic hepatitis C, but it is possible that NS5A plays a key role in HCV-induced pathogenesis.

## Supporting Information

S1 Fig Immunostaining of liver tissues of mice without LPS. Liver tissue sections derived from saline-injected transgenic (Tg) and nontransgenic (NT) mice were immunohistologically stained with an anti-phospho-STAT3 antibody.
(TIFF)

S2 Fig Specific co-immunopreciptation of IRAK1 with TRAF6 in LPS-injected nontransgenic mouse. Liver tissue lysates of NS5A-transgenic (Tg) and non-transgenic (NT) mice were immunoprecipitated with an anti-TRAF6 antibody or control IgG followed by immunoblotting with anti-IRAK1 and anti-TRAF6 antibodies.
(TIFF)

S3 Fig Interaction of MyD88 with NS5A derived from genotype 1a, 1b, and 2a. Huh7 cells were transfected with a myc-tagged MyD88 plasmid together with an HA-tagged NS5A plasmid derived from genotype 1a, 1b, and 2a. Cells were harvested after 48h and cell lysates were immunoprecipitated with an anti-HA antibody followed by immunoblotting with anti-myc and anti-HA antibodies.
(TIFF)

## Abbreviations

HCV: hepatitis C virus
HCC: hepatocellular carcinoma
NS: nonstructural
IFN: interferon
ISDR: interferon sensitivity-determining region
PKR: interferon-inducible double-stranded RNA-activated protein kinase
IL: interleukin
ISG: IFN-stimulated genes
STAT: signal transducer and activator of transcription
TLR: toll-like receptor
MyD88: myeloid differentiation factor 88
IRAK1: interleukin-1 receptor-associated kinase
TNF: tumor necrosis factor
TRAF6: tumor necrosis factor receptor associated factor-6
MAPK: mitogen-activated protein kinase
NF-κB: nuclear factor-κB
LPS: lipopolysaccharide
PCR: polymerase chain reaction
AST: aspartate aminotransferase
ALT: alanine aminotransferase
ERK: extracellular signal-regulated kinase
IRF: IFN regulatory factor
GAPDH: glyceraldehyde-3-phosphatate dehydrogenase
EMSA: electrophoretic mobility shift assay
CCL: C-C motif chemokine ligand
RANTES: regulated on activation, normal T cell expressed and secreted
2-5OAS: 2’-5’ oligoadenylate synthase
CRP: C-reactive protein
SAA1: serum amyloid A1
TAK1: transforming growth factor β-activated protein kinase-1
ALD: alcoholic liver diseases
NAFLD: non-alcoholic fatty liver diseases
