# Supplementary figures and images for: Nonstructural 5A Protein of Hepatitis C Virus Interferes with Toll-Like Receptor Signaling and Suppresses the Interferon Response in Mouse Liver
Source: PLoS One. 2017 Jan 20;12(1):e0170461. doi: 10.1371/journal.pone.0170461 (PMC5249188; doi:10.1371/journal.pone.0170461)

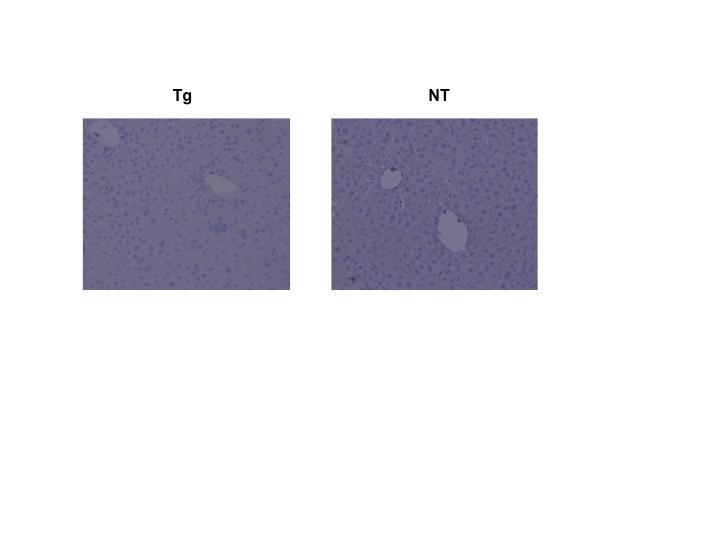

Supplement: S1 Fig — Liver tissue sections derived from saline-injected transgenic (Tg) and nontransgenic (NT) mice were immunohistologically stained with an anti-phospho-STAT3 antibody. (TIFF) [file pone.0170461.s001.tiff]

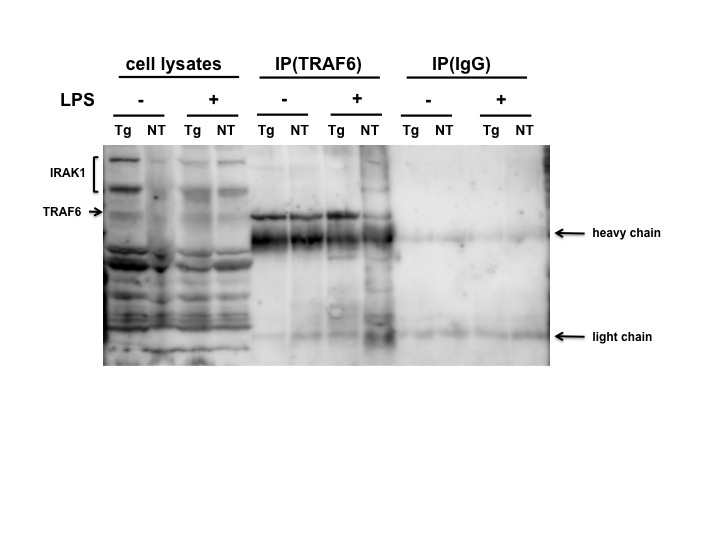

Supplement: S2 Fig — Liver tissue lysates of NS5A-transgenic (Tg) and non-transgenic (NT) mice were immunoprecipitated with an anti-TRAF6 antibody or control IgG followed by immunoblotting with anti-IRAK1 and anti-TRAF6 antibodies. (TIFF) [file pone.0170461.s002.tiff]

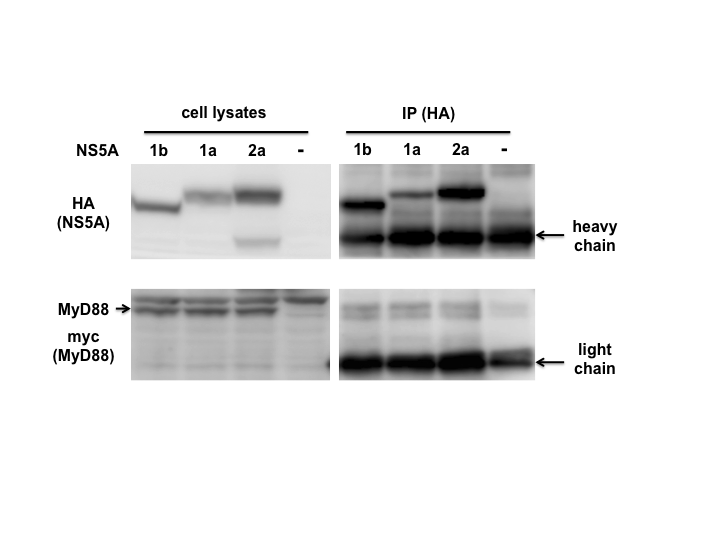

Supplement: S3 Fig — Huh7 cells were transfected with a myc-tagged MyD88 plasmid together with an HA-tagged NS5A plasmid derived from genotype 1a, 1b, and 2a. Cells were harvested after 48h and cell lysates were immunoprecipitated with an anti-HA antibody followed by immunoblotting with anti-myc and anti-HA antibodies. (TIFF) [file pone.0170461.s003.tiff]
